# Effects of Caffeine Intake on Endurance Running Performance and Time to Exhaustion: A Systematic Review and Meta-Analysis

**DOI:** 10.3390/nu15010148

**Published:** 2022-12-28

**Authors:** Ziyu Wang, Bopeng Qiu, Jie Gao, Juan Del Coso

**Affiliations:** 1 Graduate School, Beijing Sport University, Beijing 100084, China; 2 College of Swimming, Beijing Sport University, Beijing 100084, China; 3 Centre for Sport Studies, Rey Juan Carlos University, 28943 Fuenlabrada, Spain

**Keywords:** caffeine, ergogenic aid, runners, dietary supplement, exercise

## Abstract

Caffeine (1,3,7-trimethylxanthine) is one of the most widely consumed performance-enhancing substances in sport due to its well-established ergogenic effects. The use of caffeine is more common in aerobic-based sports due to the ample evidence endorsing the benefits of caffeine supplementation on endurance exercise. However, most of this evidence was established with cycling trials in the laboratory, while the effects of the acute intake of caffeine on endurance running performance have not been properly reviewed and meta-analyzed. The purpose of this study was to perform a systematic review and meta-analysis of the existing literature on the effects of caffeine intake on endurance running performance. A systematic review of published studies was performed in four different scientific databases (Medline, Scopus, Web of Science, and SportDiscus) up until 5 October 2022 (with no year restriction applied to the search strategy). The selected studies were crossover experimental trials in which the ingestion of caffeine was compared to a placebo situation in a single- or double-blind randomized manner. The effect of caffeine on endurance running was measured by time to exhaustion or time trials. We assessed the methodological quality of each study using Cochrane’s risk-of-bias (RoB 2) tool. A subsequent meta-analysis was performed using the random effects model to calculate the standardized mean difference (SMD) estimated by Hedges’ *g* and 95% confidence intervals (CI). Results: A total of 21 randomized controlled trials were included in the analysis, with caffeine doses ranging between 3 and 9 mg/kg. A total of 21 studies were included in the systematic review, with a total sample of 254 participants (220 men, 19 women and 15 participants with no information about gender; 167 were categorized as recreational and 87 were categorized as trained runners.). The overall methodological quality of studies was rated as unclear-to-low risk of bias. The meta-analysis revealed that the time to exhaustion in running tests was improved with caffeine (*g* = 0.392; 95% CI = 0.214 to 0.571; *p* < 0.001, magnitude = medium). Subgroup analysis revealed that caffeine was ergogenic for time to exhaustion trials in both recreational runners (*g* = 0.469; 95% CI = 0.185 to 0.754; *p* = 0.001, magnitude = medium) and trained runners (*g* = 0.344; 95% CI = 0.122 to 0.566; *p* = 0.002, magnitude = medium). The meta-analysis also showed that the time to complete endurance running time trials was reduced with caffeine in comparison to placebo (*g* = −0.101; 95% CI = −0.190 to −0.012, *p* = 0.026, magnitude = small). In summary, caffeine intake showed a meaningful ergogenic effect in increasing the time to exhaustion in running trials and improving performance in running time trials. Hence, caffeine may have utility as an ergogenic aid for endurance running events. More evidence is needed to establish the ergogenic effect of caffeine on endurance running in women or the best dose to maximize the ergogenic benefits of caffeine supplementation.

## 1. Introduction

Caffeine (1,3,7-trimethylxanthine) is one of the most widely consumed performance-enhancing substances in sport due to its well-established ergogenic effects in a myriad of exercise situations [1,2,3]. Caffeine’s ergogenicity is obtained in humans through several physiological mechanisms including increased central nervous system drive, increased catecholamine release, and enhanced skeletal muscle contractile capacity [4,5,6]. The benefits of caffeine in sport can also be obtained by psychobiological responses, mainly displayed as an increase in physical performance when the individual believes that they have received an ergogenic dose of caffeine, a phenomenon known as the placebo effect of caffeine [7,8]. However, there is a consensus establishing the ability of caffeine to act as an adenosine A_1_ and A_2A_ receptor antagonist as the main mechanism to explain caffeine ergogenicity during locomotor activities [9,10,11]. With this theory, the blockage of adenosine receptors with caffeine would affect the release of norepinephrine, dopamine, acetylcholine, and serotonin, among other neurotransmitters, reducing pain and perceived exertion during exercise, and delaying fatigue. This mechanism would explain the ergogenic effect of caffeine on endurance exercise [12,13], anaerobic-based exercise [14] and strength/power exercise [15], and sports with an intermittent nature [16,17].

Although the effect of caffeine to enhance endurance exercise performance was proposed several decades ago [18], and several international societies today consider caffeine as an ergogenic substance for endurance exercise [19,20], the outcomes of previous studies regarding the effect of caffeine on this topic are not unanimous, especially for running activities. Overall, it is established that consuming low-to-moderate doses of caffeine (from ~200 to ~400 mg, equivalent to 3 to 6 mg of caffeine per kg of body mass) ~1 h before exercise (15~80 min) improves endurance performance by 2~7% [12,21,22]. Most of this evidence was established with laboratory-based studies in which cycling on a cycle ergometer was the exercise activity investigated. However, in experiments about caffeine ergogenicity on endurance running performance, the benefits of this substance are less clear and there is a certain discrepancy in the studies’ outcomes [23,24,25,26].

Currently, there is only one systematic review about the effect of caffeine on endurance running performance, and the authors concluded that caffeine intake appeared to enhance runners’ athletic performance by 1.1% [22]. However, this systematic review did not include a meta-analysis to quantify the pooled effect of caffeine on running performance. Additionally, it was performed in 2013, and did not include the results of recent investigations on this topic. Hence, the purpose of this study was to perform a systematic review of the existing literature on the effects of caffeine intake on endurance running performance and subsequently apply a meta-analysis to identify the effect of caffeine on time to exhaustion and time trial runs. We hypothesized that caffeine supplementation would enhance time to exhaustion during running trials and would reduce time to complete endurance running trials with a fixed distance.

## 2. Methods Section

### 2.1. Search Strategy

This systematic review was developed following the Preferred Reporting Items for Systematic Reviews and Meta-analyses (PRISMA) 2020 statement [27]. The search terms included a mix of Medical Subject Headings (MeSH) and free-text words for key concepts related to caffeine and endurance running performance (see Appendix A). No filters or limits were used for the search. The sources of information were obtained by searching for studies using MEDLINE (via OVID), Scopus, Web of Science, and SPORTDiscus (via EBSCO). The search was established from the start of construction to 5 October 2022. The search strategy was different for each database, and it can be consulted in Appendix A. All titles and abstracts from the search were downloaded to Endnote 20 (Clarivate Analytics, London, UK) and manual cross-referencing was performed to identify duplicates and any potential missing studies. Titles and abstracts were then screened for a subsequent full-text review. The search for published studies was independently performed by two authors (Z.W. and B.Q.) and disagreements were resolved through discussion.

### 2.2. Inclusion Criteria

We defined the inclusion criteria according to PICOS principles [28]. We only incorporated in the review studies with crossover experimental designs in which the ingestion of caffeine was compared to a placebo in a single- or double-blind randomized manner and the outcomes were associated with endurance running performance. We considered any form of caffeine intake to be included in the review, but only if the effect of caffeine on endurance performance could be isolated; meanwhile, we excluded dietary supplements and foodstuffs where caffeine was provided in addition to other ergogenic aids (e.g., caffeinated energy drinks). Data from the studies in which a comparison of caffeine and placebo was made with an emphasis on placebo effects [29] or after sleep deprivation [30] were removed, as this may exaggerate or reduce the true effect of caffeine and is also inconsistent with single- or double-blind trials. We only considered samples that were categorized as healthy runners irrespective of their fitness level, and participants were excluded if they reported regular participation in other sports in addition to running (e.g., football, triathlon, etc.). Systematic reviews and meta-analyses were excluded, in addition to those original studies with no full text available, non-peer-reviewed articles, opinion pieces, commentaries, case reports and editorials. See Table 1 below for details of the inclusion criteria.

### 2.3. Data Extraction

We performed data extraction in the following topics: (a) study design; (b) sample characteristics and participants’ running experience; (c) training status; (d) caffeine dose and administration form; (e) time of ingestion with respect to exercise onset; (f) running conditions (i.e., on a standard 400 m track, road, or treadmill in laboratory conditions); and (g) endurance performance outcomes, as the time to exhaustion or time employed to complete a trial with fixed distance or amount of work. Two authors performed the data extraction separately (Z.W. and B.Q.). In instances where data were presented in a graphical format, images were enlarged to improve the precision of the data estimates and data were extracted from studies’ figures using WebPlotDigitizer [31]. In the event of disagreements between the two assessors regarding data extraction, they were resolved through discussion and consultation with a third party when necessary.

### 2.4. Risk of Bias

The Cochrane’s risk-of-bias tool (RoB 2) was used to rate the methodological quality of the included studies [32]. The seven areas covered by this tool are: random sequence generation, allocation concealment, participant and personnel blinding, outcome assessor blinding, insufficient outcome data, selective reporting of results, and other possible sources of bias. The application of RoB 2 for the included studies was performed by two separate authors (Z.W. and B.Q.), and disagreements were resolved through discussion and consultation with a third party when necessary.

### 2.5. Data Analysis

The Comprehensive Meta-Analysis software (version 3; Biostat, Englewood, NJ, USA) was used to compare the effects of caffeine vs. placebo ingestion on study outcomes using standardized mean differences (SMDs), estimated by Hedges’ *g* and 95% confidence intervals (CI). All the meta-analyses were performed using the random-effects model. We considered the SMD of ≤0.20, 0.20–0.49, 0.50–0.79, and ≥0.8 as small, medium, large and very large, respectively, based on a previous categorization [33]. The statistical significance threshold was set at *p* < 0.050. For each outcome, the SMD was calculated using mean and standard deviation values from the placebo and caffeine trials, the sample size from each study, and the correlations between the trials. Only two studies [34,35] reported correlation values for the performance data between the placebo and caffeine conditions, with a 0.87 correlation for Whalley et al. [34] and 0.86 correlation for Bell et al. [35], respectively. For the remaining studies, a 0.5 correlation was assumed, as recommended by Follmann et al. [36]. For each outcome, a minimum of three studies were required to perform the meta-analysis [37]. In studies with several caffeine–placebo comparisons, such as different doses or forms of caffeine administration, we obtained data independently for each caffeine–placebo comparison. We assumed that the sample size was the same in each separate caffeine–placebo comparison, as each participant completed all testing conditions in multi-arm studies [38]. A subgroup analysis was conducted on the effects of caffeine on endurance running performance based on the participants’ training levels (trained vs. recreational). The assignation of each study’s sample to the subgroup of trained vs. recreational runners was based on the participants’ characteristics reported in the study. For instance, when the sample was described as “elite or sub-elite”, “varsity”, “competitive” or “well-trained”, the participants were included in the subgroup of trained runners. If the sample was described as “amateur” or “recreational”, the participants were included in the subgroup of recreational runners. An additional subgroup analysis was conducted on the effects of caffeine on endurance running performance based on the distance covered during the running trial. The outcomes of running trials between 800 and 5000 m were included in the subgroup of middle-distance, and running trials covering more than 5000 m were included in the subgroup of long-distance. Finally, the magnitude of caffeine’s effect on endurance performance was presented as the percentage of change calculated by the formula proposed by Lopez-Gonzalez et al. [39].

We used the *I*^2^ statistic to assess the degree of heterogeneity, with values ≤50% indicating low heterogeneity, 50–75% indicating moderate heterogeneity, and >75% indicating high heterogeneity [40]. Potential asymmetries in funnel plots and Egger’s linear regression test were employed to detect publication bias [41].

## 3. Results

### 3.1. Search Outcomes

**Figure 1:**
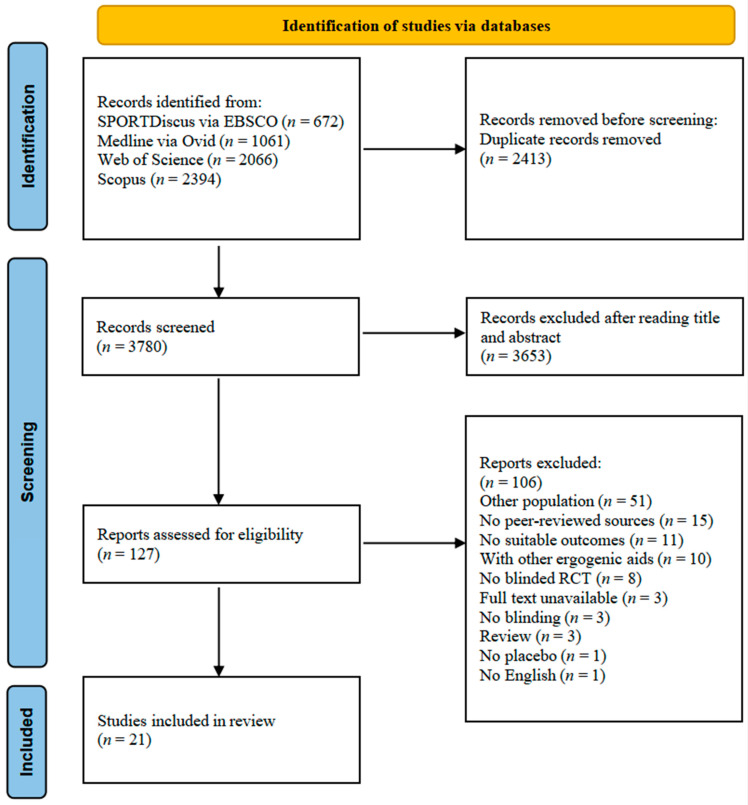
Flow diagram of literature search according to the PRISMA 2020 statement.

We obtained 6193 studies through database searching. After deleting duplicates and screening the titles and abstracts of all the remaining original papers, we evaluated 127 full-text articles for inclusion in our research. We eliminated 106 papers for various reasons, leaving only 21 studies to be included in the systematic review (Figure 1). The experiments that met the inclusion criteria were published between 1991 and 2022.

### 3.2. Study Characteristics

In the 21 studies included in this systematic review [7,23,24,25,26,30,34,35,42,43,44,45,46,47,48,49,50,51,52,53,54], there was a total sample of 254 participants, including 220 men, 19 women and 15 participants with no information about gender. The participants were all runners, of which 167 were categorized as amateur and 87 were categorized as trained runners. All studies were crossover randomized controlled trials. A total of 18 studies [7,23,24,25,26,30,35,42,43,45,46,48,49,50,51,52,53,54] provided caffeine in liquid or capsule form, with doses normalized by participants’ body mass. In these studies, the doses of caffeine administered ranged from 3 to 9 mg/kg. A total of three studies [34,44,47] provided absolute doses of caffeine in the form of caffeine powder, gum, and mouth strips with doses ranging from 200 to 300 mg. The general data of the experiments included in this systematic review are depicted in Table 2.

### 3.3. Methodological Quality of Included Studies

**Figure 2:**
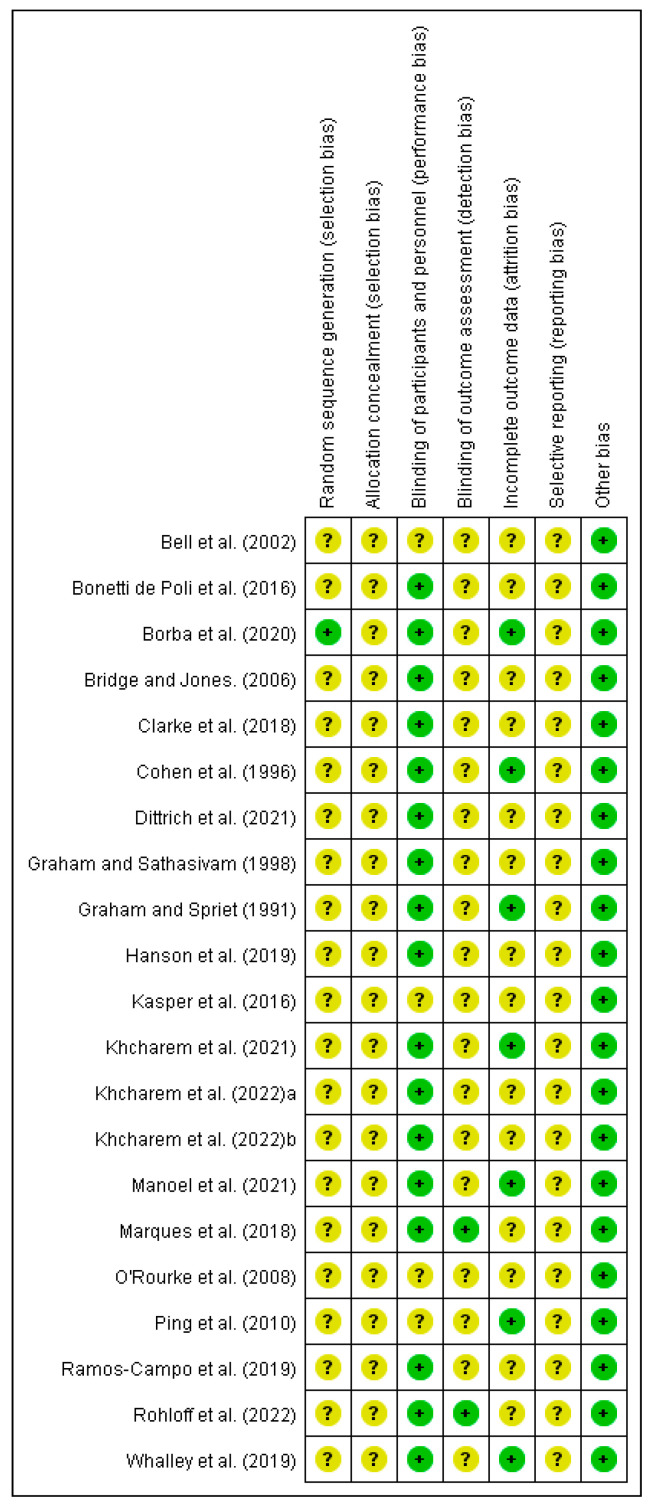
Risk of bias summary: determination of the risk of bias items for included studies. (+) = low risk of bias; (?) = unclear risk of bias. A total of 21 papers [7,23,24,25,26,30,34,35,42,43,44,45,46,47,48,49,50,51,52,53,54] were reviewed.

Figure 2 displays the categorization for each RoB 2 item for each included study. Regarding selection bias, we judged only one study [53] as low risk because it reported the appropriate method of participant randomization sequences. We defined all other studies as unclear because none provided sufficient information for this item. In addition, the allocation concealment process was not detailed in any of the studies; therefore, we rated their risk as unclear for allocation concealment. We considered that four studies were unclear regarding the blinding process of participants and researchers [35,47,50,51]. The remaining studies were all low risk for this item. Only two studies [7,26] described the blinding of outcome assessors, and they were rated as low-risk. All other studies did not provide sufficient information; therefore, they were rated as having an unclear risk. Most of the included studies [7,23,24,26,30,35,43,44,46,47,49,50,52,54] had no reported participant dropouts, and therefore they were rated unclear. None of the included studies had trial registration protocols. We identified no other sources of bias in the included studies. Thus, we categorized the overall methodological quality as with an unclear-to-low risk of bias.

### 3.4. Effects of Interventions

#### 3.4.1. Time to Exhaustion Runs

**Figure 3:**
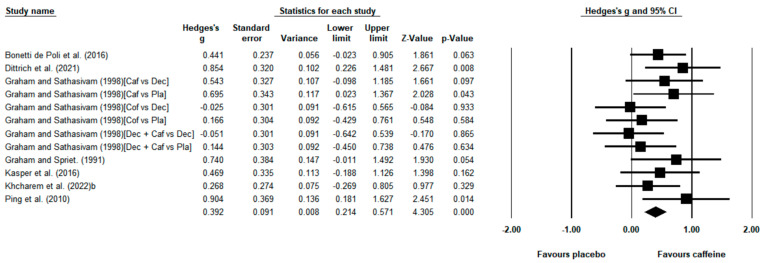
Effect of caffeine ingestion as compared to placebo on time to exhaustion runs. The forest plot shows standardized mean differences with 95% confidence intervals (CI) for 12 unique placebo–caffeine comparisons in 7 studies [43,44,45,46,47,49,51]. The diamond at the bottom of the graph represents the pooled standardized mean difference following random effects meta-analyses. A positive value reflects an increase in the time to exhaustion with caffeine with respect to the placebo. The size of the plotted squares reflects the relative statistical weight of each study. Caf, caffeine; Dec, decaffeinated coffee; Pla, placebo.

Seven studies [43,44,45,46,47,49,51] including 12 placebo–caffeine comparisons investigated the effect of caffeine on time-to-exhaustion runs. Pooled data from these investigations showed that the caffeine intake was effective in prolonging the time to exhaustion compared with the placebo (*g* = 0.392; 95% CI = 0.214 to 0.571; *p* < 0.001, magnitude = medium; Figure 3). We observed no significant heterogeneity (Q = 11.436; *df* = 11; *p* = 0.408; *I*^2^ = 3.809%). Overall, caffeine increased time to exhaustion during running trials by 16.97% ± 14.65%. The subgroup analysis of training status revealed that caffeine increased time to exhaustion in recreational runners (*g* = 0.469; 95% CI = 0.185 to 0.754; *p* = 0.001, magnitude = medium; *I*^2^ = 0%) and trained runners (*g* = 0.344; 95% CI = 0.122 to 0.566; *p* = 0.002, magnitude = medium; *I*^2^ = 22.44%). Funnel plots showed evidence of non-publication bias, which was confirmed by Egger’s linear regression test (*p* > 0.050; Appendix B).

#### 3.4.2. Endurance Time Trial Performance

**Figure 4:**
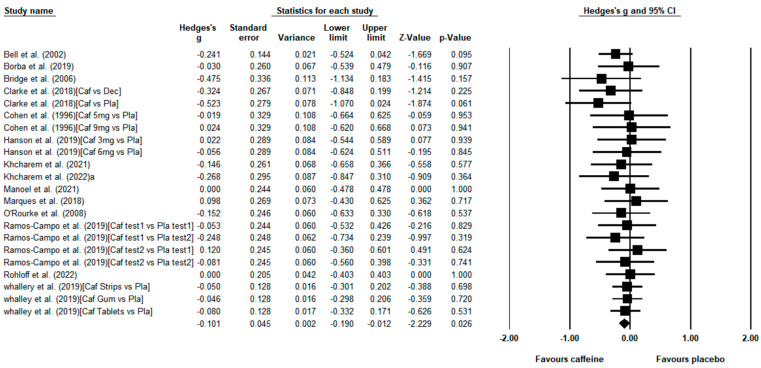
Effect of caffeine ingestion as compared to placebo on endurance running time trials. The forest plot shows standardized mean differences with 95% confidence intervals (CI) for 22 unique placebo–caffeine comparisons in 14 studies [7,23,24,25,26,30,34,35,42,48,50,52,53,54]. The diamond at the bottom of the graph represents the pooled standardized mean difference following random effects meta-analyses. A negative value reflects a reduction in the time employed to complete a given distance with caffeine with respect to the placebo. The size of the plotted squares reflects the relative statistical weight of each study. Caf, caffeine; Dec, decaffeinated coffee; Pla, placebo.

Fourteen randomized controlled trials [7,23,24,25,26,30,34,35,42,48,50,52,53,54] including 22 placebo–caffeine comparisons evaluated the effect of caffeine on endurance running performance in time trials. The meta-analysis revealed a significant reduction in the time employed to complete the running trials with caffeine vs. placebo (*g* = −0.101; 95% CI = −0.190 to −0.012, *p* = 0.026, magnitude = small; Figure 4), and this meta-analysis did not contain substantial heterogeneity (Q = 8.580; *df* = 21; *p* = 0.992; *I*^2^ = 0%). The average reduction in time during endurance running events was −0.71% ± 0.83%. However, the subgroup analysis indicated that caffeine failed to improve endurance running performance in time trials carried out by trained runners (*g* = −0.140; 95% CI = −0.295 to 0.014, *p* = 0.075) and by recreational runners (*g* = −0.082; 95% CI = −0.190 to 0.027, *p* = 0.141; Table 3). The subgroup analysis of distance also revealed that caffeine was not ergogenic in middle-distance or long-distance time trials (Table 3). A funnel plot revealed no indication of publication bias, which was confirmed by Egger’s linear regression test (*p* > 0.05; Appendix B).

## 4. Discussion

Caffeine supplementation is habitually recommended as an ergogenic aid for endurance performance, including endurance running [19,20]. However, most of the evidence on caffeine’s ergogenicity in aerobic-based scenarios is based on studies with cycling trials [12]. Although the basics of caffeine’s ergogenicity can be transferred from endurance cycling to endurance running (e.g., minimal effective dose, the timing of ingestion, habituation, etc.), the particularities of a footrace may mean that the magnitude of the ergogenic effect is different in running than in cycling. A recently published review suggested that caffeine supplementation in the form of coffee improved endurance performance by 3.1% [55]. However, this review included a pool of cycling and running trials, and it is difficult to ascertain if the ergogenic effect of caffeine is of similar magnitude for cycling and running. Interestingly, the effectiveness of caffeine in enhancing endurance running performance has not been properly summarized, despite there being plenty of studies on this topic to accurately determine the existence (or not) and magnitude of the ergogenic effect of caffeine on endurance running. Hence, the aim of this systematic was to analyze the existing literature on the effects of caffeine intake on endurance running performance and subsequently apply a meta-analysis to identify the effect of caffeine on time to exhaustion and time trial runs. The main results of this systematic review indicate that caffeine intake in doses ranging from 3 to 9 mg per kg of body mass (1) enhanced time to exhaustion during running trials with an effect of medium magnitude and an overall increase of 16.97% ± 14.65% and (2) produced a small but statistically significant effect on endurance running time trial protocols with a mean reduction of −0.71% ± 0.83%. Collectively, all this information supports the recommendation of caffeine intake for endurance runners, but now with the meta-analytical support of specific studies in which caffeine’s benefits were tested in endurance running protocols. Nevertheless, caffeine seems more ergogenic for time-to-exhaustion runs than for time trials, suggesting that this substance may be more useful for prolonged running events where time to exhaustion is a performance factor.

### 4.1. Effect of Caffeine Intake on Time to Exhaustion Runs

Both time-to-exhaustion and time-trial exercise test protocols are commonly used to examine the influence of experimental interventions on endurance performance. In the context of endurance running, time-to-exhaustion protocols represent an unusual scenario with respect to real-environment running [22], as most endurance running events consist of completing a given distance as fast as possible. However, time-to-exhaustion runs provide a measure of an athlete’s endurance capacity and they are considered a valid measure of endurance performance [56]. In the current systematic review, seven studies, including 12 unique placebo–caffeine comparisons, tested the effect of caffeine on running protocols that entailed participants performing submaximal intensity running until they could no longer maintain the required speed. Overall, the values of time to exhaustion were higher with caffeine than in the placebo situation, and the meta-analysis revealed a statistically significant effect of caffeine in this performance variable (Figure 3). Caffeine promotes the production of ß-endorphins and dopamine [57], which can lessen perceived effort and discomfort [58,59]. These factors can explain the ergogenic effect of caffeine on time to exhaustion runs, as the “exhaustion time” when runners decide to stop running is associated with feelings of fatigue and muscle pain. Although running events consisting of “run as long as you can” are scarce, the higher time to exhaustion induced by caffeine may apply to ultra-endurance running events where athletes are required to run in scenarios close to exhaustion, such as 24 h races and 100-mile races or mountain running events. It is important to note that performance in time-to-exhaustion trials is habitually more variable than in time trials, especially for those with a lower training background [60,61]. However, our subgroup analyses indicated that caffeine ingestion significantly increased run-to-fatigue time to a moderate degree in both recreational and trained runners. In summary, it seems safe to conclude that caffeine is an effective substance to enhance running performance in situations that entail running until volitional fatigue.

### 4.2. Effect of Caffeine Intake on Endurance Running Time Trials

The use of time trials offers a better scenario to study the effect of caffeine on endurance running performance, as runners’ performance is more reproducible in time-trial runs than in running to exhaustion events [56]. This likely explains why 14 studies used time trials while only seven studies used time-to-exhaustion runs to determine the effect of caffeine on running performance. The meta-analysis revealed that caffeine has a small effect but significant ergogenic effect to reduce time in endurance running for a given distance (Figure 4). The ergogenic effect of caffeine on running time trials was lower compared to the effect of this substance on time-to-exhaustion runs (0.71% ± 0.83% vs. 16.97% ± 14.65%, respectively). The explanation for the different magnitude of caffeine’s benefits on these two types of endurance performance running trials may be associated with the characteristics of each running protocol. In time-to-exhaustion runs, the intensity is fixed, and runners cannot adjust the pace during the running trial. However, in time trials, like in real running races, runners may adjust their pace depending on their feelings of fatigue and the distance left to complete the trial [62]. As an adenosine antagonist [5], caffeine can decrease fatigue by crossing the blood–brain barrier and inhibiting A_1_ and A_2_ monoadenosine receptors [5,9]. So, caffeine may have an inhibitory effect on perceptual response during exercise, which may give participants an increased ability to tolerate the discomfort associated with fatigue during exercise, effectively masking the sensation of fatigue [58]. Therefore, caffeine intake may produce a faster running speed than placebo intake with the same perceptual response [63], thereby influencing running pacing strategies [64]. Independently of the type of test employed, caffeine produced statistically significant benefits for both time-to-exhaustion runs and time trials, suggesting that caffeine is a substance with the potential for enhancing performance in different endurance running scenarios.

Interestingly, when analyzing the ergogenic effect of caffeine on time-trial runs according to participants’ training status, the benefit of caffeine was not statistically significant for trained runners or recreational runners (Table 3). Previous studies indicated that caffeine is more effective in reducing the time to complete specific running distances in trained athletes, and trained runners are more reliable [60,61]. Similar results have been found in other types of exercise, such as swimming, as it has been suggested that caffeine has a facilitative effect on trained but not “active” swimmers [65]. Although more studies are granted on this topic, we can assume that training status is not a modifying factor for the ergogenic effect of caffeine on endurance performance. Likewise, when considering the distance of the running time trials, caffeine did not reduce the time to complete middle-distance or long-distance trials (Table 3), despite it previously being suggested that caffeine is more effective in long endurance events than in short but intense endurance events [38,66]. Overall, the effect of caffeine on improving running time trial performance was statistically significant but small, which demonstrated that the potential ergogenic effect of caffeine was the same across subgroups of training status and distance.

### 4.3. Limitations and Future Lines of Research

Although we only selected blinded randomized controlled studies, the placebo choice appears to be diverse in some studies (e.g., sugar-free, sugary, and decaffeinated), and caffeine was also taken with other compounds (e.g., carbohydrates and artificial sweeteners). Thus, it is possible that the actual effects of caffeine may be masked or exaggerated by some of these substances. In addition, different sources of caffeine (capsules, tablets, gums, beverages, etc.) may affect the pharmacokinetics of caffeine [11], thus influencing the results of different studies. Another limitation is that, due to the differences in participants’ characteristics and form of caffeine administration, it was not possible to meta-analyze the effect of participants’ sex, form of caffeine intake or caffeine dose on the benefits obtained with caffeine supplementation. In this regard, only 7.5% of the pooled sample of this systematic review were women, suggesting that the outcomes of this study possess a sex bias. For this reason, it is perhaps more accurate to indicate that caffeine’s ergogenicity on endurance running performance in men is supported by the results of this study, while the benefits of this substance for women endurance runners should be further investigated. Last, in the current analysis, we only focused on running performance variables, namely the time to exhaustion and the time to complete a given distance. The study of the effect of caffeine on physiological and perceptual responses during endurance running such as heart rate, blood lactate concentration and ratings of perceived fatigue requires further investigation and analysis, as these responses may contribute to explaining the ergogenic effect of caffeine. From a methodological perspective, it should be mentioned that most of the studies were classified as having low risk of bias, as they all utilized randomized crossover designs. Therefore, the outcomes of the current study are not influenced by the inclusion of studies with poor methodological quality, which strengthens the main conclusions.

## 5. Conclusions

Pre-exercise caffeine supplementation, in the range of 3 to 9 mg per kg of body mass, showed a medium-size ergogenic effect to increase the time to exhaustion in running trials and a small-size effect to improve performance in running time trials. These outcomes confirm the utility of caffeine as an ergogenic aid for endurance running events where time to exhaustion is key for performance or in competitions with a fixed distance. Still, the evidence is not enough to determine whether the ergogenic effect of caffeine is of similar magnitude in men and women, as women only represented a minor portion of the study participants in this systematic review. Further investigation is also needed to establish the minimal effective dose of caffeine, the existence of tolerance to caffeine’s ergogenicity with chronic habituation, and the most common drawbacks of caffeine supplementation in the context of endurance running.

## Figures and Tables

**Table 1:** PICOS criteria for the inclusion of crossover experimental designs in which the ingestion of caffeine was compared to a placebo in a single- or double-blind randomized manner. For inclusion, the study outcomes should be associated with endurance running performance.

Parameters	Inclusion	Exclusion	Extraction
P	Healthy runners	Participants of other sports disciplines or non-athletic population	Number of participants, gender, training level and experience, age, daily caffeine intake, and anthropometric characteristics
I	Caffeine	Caffeine intake in combination with other ergogenic aids	Dosage, the form of administration, and timing of ingestion
C	Placebo	Trials without a true placebo/control situation	Components, dosage, the form of administration
O	Endurance running performance	Any other form of endurance exercise different from running	Time to exhaustion trials and time trials
S	Single- or double-blind randomized controlled trials published in peer-reviewed journals	Systematic reviews, conference abstracts, graduate student dissertations, and editorials	Experimental design, date of publication

**Table 2:** Detailed characteristics of included trials (*n* = 21).

Author(s) Year Country	N (Male/Female) Age Body Mass	Running Experience	Caffeine Administration	Timing	Comparator	Running Conditions	Measures	Running Tests
Bell et al., 2002 Canada [35]	12 (10/2) 33 ± 8 years 75 ± 11 kg	Recreational	4 mg/kg in capsules	60 min- pre-exercise	300 mg dietary fiber capsules	Treadmill	10 km time ^NS^	TTP
Borba et al., 2019 Brazil [53]	13 (8/5) 28.46 ± 5.63 23.58 ± 3.90 kg/m^2^	Recreational	6 mg/kg in hot water	60 min pre-exercise	Hot water	Track (400 m)	1600 m runs ^NS^	TTP
Bonetti de Poli et al., 2016 Brazil [43]	18 (18/0) 29 ± 7 years 72.1 ± 5.8 kg	Recreational	6 mg/kg in capsules	60 min pre-exercise	Dextrose capsules	Treadmill	TTE test ↑	TTE
Bridge and Jones 2006 United Kingdom [54]	8 (8/0) 21.3 ± 1.2 years 69.3 ± 5.0 kg	Trained	3 mg/kg in capsules	60 min pre-exercise	Glucose capsules	Track (400 m)	8 km time ↑	TTP
Clarke et al., 2018 United Kingdo [23]	13 (13/0) 24 ± 6 years 69.3 ± 4.7 kg	Trained	0.09 g/kg coffee (including 3 mg/kg)	60 min pre-exercise	1. Decaffeinated coffee in warm water 2. Warm water with coffee flavor and color	Indoor 200 m running track with banked curves	mile race ↑	TTP
Cohen et al., 1996 United States of America [42]	7 (5/2) 33.29 ± 9.18 years NI	Trained	1. 5 mg/kg in capsules 2. 9 mg/kg in capsules	60 min pre-exercise	Baking flour capsules	Outdoor road	21 km runs ^NS^	TTP
Dittrich et al., 2021 Brazil [44]	12 (12/0) 31.3 ± 6.4 years 70.5 ± 6.6 kg	Trained	300 mg in chewing gum	immediately pre-exercise	Noncaffeinated chewing gum	Treadmill	TTE test ↑	TTE
Graham and Spriet 1991 Canada [45]	7 (6/1) 28.3 ± 5.63 years 67.2 ± 8.33 kg	Trained	9 mg/kg in capsules	60 min pre-exercise	9 mg/kg dextrose capsules	Treadmill	TTE test ↑	TTE
Graham and Sathasivam 1998 Canada [46]	9 (8/1) 21.1 ± 6.5 years 73.1 ± 5.5 kg	Recreational	1. 4.5 mg/kg in capsules 2. regular coffee (including 4.5 mg/kg) 3. decaffeinated coffee plus 4.5.mg/kg in capsules	60 min pre-exercise	1. decaffeinated coffee 2. dextrose capsules	Treadmill	TTE test ↑	TTE
Hanson et al., 2019 USA [24]	10 (6/4) 26 ± 9 years 72.1 ± 8.7 kg	Trained	1. 3 mg/kg in a flavored water-based drink 2. 6 mg/kg in a flavored water-based drink	60 min pre-exercise	Flavored water-based drink without caffeine	Treadmill	10 km runs ^NS^	TTP
Kasper et al., 2016 Tunisia [47]	8 (8/0) 22 ± 2 years 70.8 ± 8.1 kg	Recreational	200 mg in capsules/ + carbohydrate mouth rinse	immediately pre-exercise	placebo capsules + carbohydrate rinse	Treadmill	TTE test ↑	TTE
Khcharem et al., 2021 Tunisia [48]	13 (13/0) 21.3 ± 0.8 years 66.5 ± 7.8 kg	Recreational	3 mg/kg in capsules	90 min pre-exercise	3 mg/kg sucrose capsules	Track (400 m)	3 km runs ↑	TTP
Khcharem et al. a 2022 Tunisia [30]	10 (10/0) 21.7 ± 1.1 66.7 ± 8.7 kg	Recreational	5 mg/kg in capsules	90 min pre-exercise	NI capsules	Track (400 m)	8 km runs ↑	TTP
Khcharem et al. b 2022 Tunisia [49]	12 (12/0) 21.7 ± 0.9 years 64.4 ± 9.4 kg	Recreational	6 mg/kg in capsules	90 min pre-exercise	6 mg/kg sucrose capsules	Track (400 m)	TTE test ↑	TTE
Manoel et al., 2021 Brazil [25]	15 (15/0) 25.2 ± 2.8 years 79.9 ± 7.7 kg	Recreational	6 mg/kg in capsules	60 min pre-exercise	Empty capsules	Track (400 m)	10 km runs ^NS^	TTP
Marques et al., 2018 Brazil [26]	12 (12/0) 23.50 ± 3.94 years 70.38 ± 8.41 kg	Recreational	5.5 mg/kg in soluble coffee	60 min pre-exercise	Decaffeinated coffee	Track (400 m)	800 m run ^NS^	TTP
O’Rourke et al., 2008 Australia [50]	15 (NI) 32.2 ± 8.8 years 68.9 ± 6.1 kg	Trained	5 mg/kg in tablets	60 min pre-exercise	5 mg/kg sugar tablets	Track (400 m)	5 km run ↑	TTP
Ping et al., 2010 Malaysia [51]	9 (9/0) 25.4 + 6.9 years 57.6 + 8.4 kg	Recreational	5 mg/kg in capsules	60 in pre-exercise	5 mg/kg placebo capsules	Treadmill	TTE test ↑	TTE
Ramos-Campo et al., 2019 Spain [52]	15 (15/0) 23.7 ± 8.2 years 64.6 ± 9.8 kg	Trained	6 mg/kg in capsules	60 min pre-exercise	6 mg/kg sucrose capsules	Track (400 m)	800 m run ^NS^	TTP
Rohloff et al., 2022 Brazil [7]	22 (22/0) 25.5 ± 8.4 years 75.0 ± 7.1 kg	Recreational	4 mg/kg in capsules	60 min pre-exercise	4 mg/kg maltodextrin capsules	Treadmill	4 km runs ^NS^	TTP
Whalley et al., 2019 New Zealand [34]	14 (10/4) 40 ± 8 years 69 ± 11 kg	Recreational	1. 200–300 mg in chewing gum 2. 200–300 mg in tablets 3. 200–300 mg in mouth strips	15 min pre-exercise	300 mg glucose powder capsule	Road	5 km runs ↑	TTP

TTP time trial performance; TTE, time to exhaustion; NI, not informed; NS, a no statistically significant difference between caffeine condition and placebo condition; ↑, a statistically significant difference between caffeine condition and placebo condition indicating an ergogenic effect of caffeine.

**Table 3:** Subgroup analysis by training status and distance on the effect of the caffeine ingestion on endurance running time trials.

Subgroup	Grouping Criteria	Number of Studies	*g*	95%CI	*p*	*I* ^2^
Training status	Recreational	10	−0.082	[−0.190, 0.027]	0.141	0%
	Trained	12	−0.140	[−0.295, 0.014]	0.075	0%
Distance	Middle-distance	14	−0.083	[−0.187, 0.020]	0.114	0%
	Long-distance	8	−0.151	[−0.324, 0.023]	0.089	0%

## Data Availability

The data presented in this study are available on request from the corresponding author (J.G.).

## Appendix A. Search Strategies Used for the Four Databases Used in the Investigation

**MEDLINE (via OVID).**

1	(Caffedrine or caffeine or Coffeinum or coffee or caffein*).ab,ti	46,031
2	Coffee/	8042
3	Caffeine/	24,875
4	1 or 2 or 3	51,853
5	Marathon Running/ or Running/	22,700
6	(run or runner or running or marathon).ab,ti.	163,459
7	(endurance or aerobic).ab,ti.	127,679
8	5 or 6 or 7	287,903
9	4 and 8	1061

**SPORTDiscus (via EBSCO).**

S1	AB caffein* OR AB coffee OR AB coffeinum OR AB caffedrine	4953
S2	AB run OR AB runner OR AB running OR AB marathon	96,403
S3	AB endurance OR AB aerobic	40,490
S4	S2 OR S3	129,153
S5	S1 AND S4	672

**Web of science.**

#1	(TS=(endurance)) OR TS=(aerobic)	211,671
#2	TS=(run) OR TS=(runner) OR TS=(running) or TS=(marathon)	675,833
#3	TS=(caffein*) OR TS=(coffee) OR TS=(coffeinum) OR TS=(caffedrine)	78,451
#4	#1 OR #2	871,860
#5	#3 AND #4	2066

**Scopus.**

(TITLE-ABS-KEY (run) OR TITLE-ABS-KEY (runner) OR TITLE-ABS-KEY (running) OR TITLE-ABS-KEY (marathon) OR TITLE-ABS-KEY (endurance) OR TITLE-ABS-KEY (aerobic)) AND (TITLE-ABS-KEY (coffee) OR TITLE-ABS-KEY (coffeinum) OR TITLE-ABS-KEY (caffedrine) OR TITLE-ABS-KEY (caffeine))	2394

## Appendix B. Results of the Egger’s Linear Regression Test

**Table:**

Outcomes	Coef.	SE	95% CI	t	*p*	Number of Trials
Time to Exhaustion	0.856	1.69	−2.90 to 4.61	0.51	0.622	12
Running Time Trials	−0.740	0.72	−2.24 to 0.762	−1.03	0.317	22
